# Targeting APP/AICD in Down syndrome

**DOI:** 10.18632/oncotarget.18860

**Published:** 2017-06-30

**Authors:** Sandra Guidi, Fiorenza Stagni, Renata Bartesaghi

**Affiliations:** Department of Biomedical and Neuromotor Sciences, University of Bologna, Bologna, Italy

**Keywords:** Down syndrome, intellectual disability, pharmacotherapy, amyloid precursor protein, amyloid precursor protein intracellular domain

## The discovery

**Figure 1:**
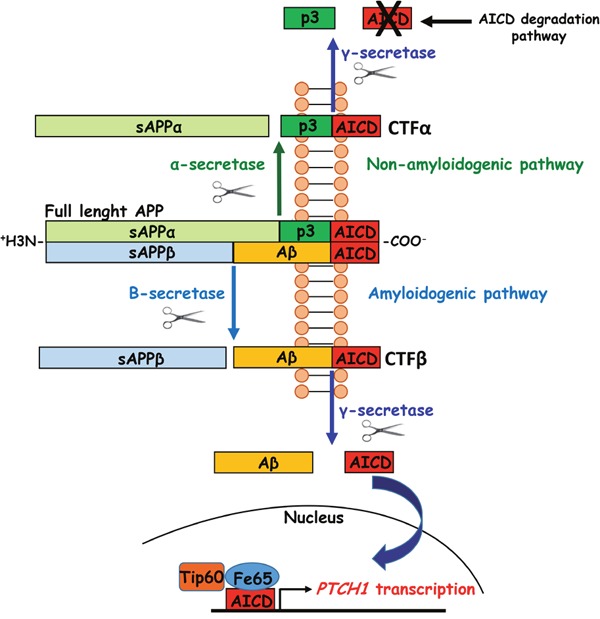
Schematic representation of APP processing and role of its product AICD in the upregulation of *PTCH1* expression

Down syndrome (DS), a genetic disorder caused by triplication of chromosome 21, is unavoidably associated with intellectual disability due to early-occurring alterations of brain development. No therapies currently exist for intellectual disability in DS. Although various triplicated genes may play a role in impairing brain development, accumulating evidence suggests that *APP* (amyloid precursor protein) is a particularly crucial gene. Cleavage of APP gives origin to AICD (amyloid precursor protein intracellular domain) a peptide that migrates to the nucleus and modifies the transcription of various genes, including *PTCH1* (*PATCHED 1*) (Figure 1). PTCH1 is a receptor of SHH (Sonic Hedgehog) that binds to a second receptor, SMO (SMOOTHENED), thereby inhibiting the activity of the SHH pathway. Increased levels of AICD due to APP triplication may enhance PTCH1 transcription, which leads to excessive inhibition of the SHH pathway. Fetuses with DS exhibit increased PTCH1 levels in the VZ/SVZ [1], suggesting that early overinhibition of the SHH pathway may be a key factor underpinning the neurogenesis reduction that characterizes DS. Prompted by this line of reasoning, we exploited the Ts65Dn mouse model of DS in order to obtain evidence regarding this issue. We found that neural precursor cells (NPCs) from the SVZ of the Ts65Dn model exhibited increased levels of APP, AICD and PTCH1. Silencing of PTCH1 restored proliferation of trisomic NPCs [1], supporting the idea that SHH pathway derangement may be a central factor underlying neurogenesis reduction in DS. We then moved to *in vivo* experiments with the intention of pharmacologically correcting AICD-mediated PTCH1 overexpression and, consequently, restoring proliferation. AICD derives from the cleavage of CTFs (carboxyterminal fragments) of APP operated by the enzyme gamma-secretase. Since reducing APP overexpression *in vivo* is unfeasible, we sought to reduce AICD formation by inhibiting the activity of gamma-secretase. To this purpose, we used ELND006, a selective inhibitor of APP gamma-secretase created by ELAN Inc [2]. We found that Ts65Dn mice treated in the first two postnatal weeks (the period of maximum neurogenesis in the hippocampal dentate gyrus) exhibited normalized hippocampal levels of PTCH1, fully restored proliferation potency in the dentate gyrus and restored total number of granule neurons [3]. One month after treatment cessation the number of proliferating cells and total number of granule neurons were still in their restored state and the dentate gyrus-CA3 connections were functionally restored [4].

## The pros and the cons

This evidence shows that neonatal treatment with an inhibitor of gamma-secretase restores hippocampal development in a mouse model of DS. It seems relevant to note that the pro-proliferative effect of treatment also involved NPCs from the SVZ [3], a germinal niche that, in conjunction with the embryonic VZ, gives origin to cortical neurons. An obvious question is now: can we envisage a translational impact for DS? The problem of pharmacological interventions is that they are, in most cases, Janus-faced. The results summarized above represent the “good face” of Janus but the “bad face” deals with the far-from-irrelevant issue of safety. We found that a short treatment with a dose of 30 mg/kg did not cause adverse effects on animals’ well-being, but in the one-month period after treatment cessation there was an increase in the mortality rate. However, a lower dose (20 mg/kg) was effective and well-tolerated. ELND006 has been created as potential therapy for Alzheimer’s disease because it reduces the formation of Abeta (that derives, in conjunction with AICD, from the cleavage of beta-CTFs). Unfortunately, a clinical trial with ELND006 had to be interrupted due to toxicity problems [5]. Therefore, our results can be regarded as proof of principle demonstration that it is possible to reinstate neurogenesis in DS by reducing AICD levels.

## The APP/AICD system as key target for the improvement of neurodevelopmental alterations in Down syndrome: a challenge for future research

What should be done next? A challenging issue is the creation of new molecules that, while selectively inhibiting APP gamma-secretase, are truly devoid of side effects. AICD derives from the cleavage of both alfa- and beta-CTFs, but the amyloidogenic cleavage pathway of APP is predominantly responsible for AICD-mediated nuclear signaling [6]. Thus, inhibitors of BACE1 (beta-site APP-cleaving enzyme 1) [6], the enzyme that cleaves APP, giving origin to beta-CTFs, or antibodies against BACE1 [7] may be exploited in order to reduce the formation of beta-CTFs and, consequently, of AICD. It has been shown that an anti-Abeta immunotherapeutic approach counteracts Abeta-related pathology in a mouse model of DS [8]. Thus, a vaccine against AICD could be created and used in order to inhibit its activity and, thus, its negative effects. Finally, drugs may be used that hamper AICD nuclear translocation. These approaches would circumvent the difficulties of identifying safe inhibitors of gamma-secretase.

DS is a relatively high incidence (1:850/1000) pathology and, thanks to an improvement in medical care, life expectancy of individuals with DS has greatly increased. Thus, the necessity for means that improve brain development is increasingly pressing. During the past decade, our group and other laboratories have been working intensely in the search of potential pharmacotherapies for DS. We believe that therapies targeted to the APP/AICD system have a strong rationale basis and sincerely hope that our discovery may spur the search for safe drugs that reinstate the functionality of this system.
